# Protective Effect of Hepatitis B Vaccine Combined with Two-Dose Hepatitis B Immunoglobulin on Infants Born to HBsAg-Positive Mothers

**DOI:** 10.1371/journal.pone.0026748

**Published:** 2011-10-28

**Authors:** Huaibin Zou, Yu Chen, Zhongping Duan, Hua Zhang

**Affiliations:** 1 Artificial Liver Center, Beijing YouAn Hospital, Capital Medical University, Beijing, China; 2 Department of Obstetrics and Gynecology, Beijing YouAn Hospital, Capital Medical University, Beijing, China; Tulane University, United States of America

## Abstract

**Background:**

Despite the use of hepatitis B (HB) vaccine and hepatitis B immunoglobulin (HBIG), a portion of infants are still non- or low-responders, or even immunoprophylaxis failure. We aimed to determine the immune response in the infants from the mothers being positive for hepatitis B surface antigen (HBsAg), by which the infants received three doses of HB vaccine in combination with two-dose 200 IU HBIG injections.

**Methods:**

In this retrospective study, 621 infants from HBsAg-positive mothers in Beijing YouAn Hospital between January 2008 and December 2009 were included. All the infants were given three doses of 10 µg HB vaccine (at 0, 1 and 6 months of age) and two-dose of 200 IU HBIG (at birth and in 2 weeks of age). Serum HBsAg and antibody to HBsAg (anti-HBs) in all the infants were determined at 7 months of age.

**Results:**

Of the 621 infants, 2.9% were immunoprophylaxis failure (positive for HBsAg), 1.4% were non-responders (anti-HBs undetectable), 95.7% were responders. The 594 responders could be categorized into three subsets, 22 were 10 to 99 IU/L for anti-HBs levels, 191 were 100 to 999 IU/L, and 381 were ≥1000 IU/L. The immunoprophylaxis failure rate was at 0% and 5.2% for the infants of HBeAg-negative and HBeAg-positive mothers(P<0.001). Infants from mothers with detectable HBV DNA had higher incidence of immunoprophylaxis failure than those of mothers without detectable HBV DNA (P = 0.002). The factors including gender, birth weight, gestation weeks, the rates of maternal HBeAg-positive, and detectable HBV DNA did not contribute to the no response to HB vaccination.

**Conclusions:**

Through vaccination by three doses of HB and two-dose of HBIG, majority of the infants (95.7%) achieved a protective level of anti-HBs at 7 months of age. Maternal HBeAg-positive and HBV DNA detectable were associated with the immunoprophylaxis failure, but not contribute to the non- or low-response to HB vaccination.

## Introduction

Chronic HBV infection is still prevalent worldwide, and it is a major cause of liver-related morbidity and mortality [1]–[3]. About 15–25% of the HBV infected patients could eventually develop cirrhosis, liver failure, or hepatocelluar carcinoma, (HCC) later in their life.

Passive and active immunizations are the most effective measures to prevent HBV infection and its consequences. For the babies from HBsAg positive mothers, use of HB vaccine and HBIG after 12 hour of birth tremendously reduces the HBV infection rate [4]–[6]. However, despite the administration, the incidence of non-responders or low-responders, even immunoprophylaxis failure still remains [7]–[10].

Previous studies have demonstrated that combination of both passive and active vaccination by HB and HBIG is superior significantly to the sole vaccination with either HB or HHIG to reduce hepatitis B occurrence [7], [11]. However, in most of the previous studies, only one dose of HBIG was used. To date, the large scale study to evaluate the immune effect of two-dose HBIG plus three doses of recombinant HB vaccine in infants of HBsAg-positive mothers has not been described Therefore, the aim of this study was to investigate the immune response and protective efficacy by a combination of two-dose HBIG and three doses of recombinant HepB vaccine for infants of HBsAg-positive mothers.

## Methods

### Study Population

In this retrospective study, newborn infants of HBsAg positive mothers were included, and all the mothers were consecutively hospitalized in the Department of Obstetrics and Gynecology in Beijing YouAn Hospital, Capital Medical University, from January 2008 to December 2009. Complete medical records were analyzed for mothers and infants. All mothers were confirmed as chronic HBV infectants.

**Figure 1:**
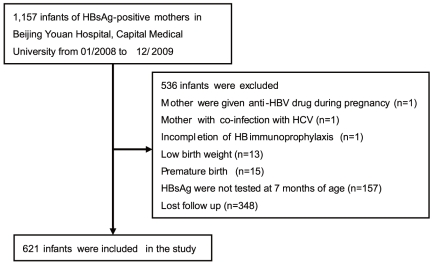
Flow chart of the participants enrolled in the study. A total of 1157 infants of HBsAg-positive mothers from January 2008 to December 2009 in Beijing Youan Hospital were reviewed. Of the 1157 infants, 536 were excluded. Thus, 621 infants were included and analyzed in the study.

Mothers with one of the following situations were excluded: 1) were given anti-viral, or immune-modifying therapy during pregnancy; 2) co-viral infection; 3) any immunologically compromised conditions. Infants exclusion criteria were as follows: 1) with low birth weight; 2) with premature birth; 3) incompletion of passive-active HB immunoprophylaxis; 4) HBsAg were not tested at 7 months of age or lost of follow-up. In the total 1,157 potential infant participants, 536 were excluded due to the various reasons mentioned above, and therefore 621 infants were included in the final analysis. The flow chart of the participants enrolled in the study was summarized in Figure 1.

This study was approved by the Institutional Review Board (IRB) of Beijing YouAn Hospital, Capital Medical University. The informed consent was waived by the IRB because this study was a retrospective assessment.

### Immunization Schedule

All infants received two doses (at birth and 2 weeks of age) of HBIG (Chengdu Institute of Biological Products, China; Hualan Biological Engineering Inc., China) injection, 200 IU (1.0 ml) each and three doses (in 0, 1, and 6 months schedule)10 µg (0.5 ml) of recombinant HB vaccine (hansenula yeast vaccine, Dalian Hissen Bio-pharm Co., China). The first HBIG and HB vaccine were given within 6 hours of birth.

The infants were consecutively followed-up in the Department of Obstetrics and Gynecology in Beijing YouAn Hospital, Capital Medical University, from August 2008 to June 2010. The serum HBV-markers (HBsAg, anti-HBs, HBeAg, anti-HBe and anti-HBc) of all infants were measured at 7 months of life.

### Laboratory Examination

In 65.5% (407/621) of mothers, the HBV markers were detected using chemiluminescent microparticle immunoassay (CMIA) kits (Abbott Diagnostics, USA), in 25.1% (156/621) of mothers, HBV markers were detected using electrical chemiluminence immunoassay kits (Roche Laboratories, Germany), and 9.3% (58/621) mothers by enzyme-linked immunosorbent assay (ELISA) kits (Beijing Wantai Biological Pharmacy Enterprise Co., China) at the central laboratory of Beijing YouAn Hospital, Capital Medical University. All the infants' HBV markers were measured by CMIA kits at the central laboratory of Beijing Youan Hospital, Capital Medical University.

The serum HBV DNA levels in pregnant women were tested by real-time quantitative Polymerase Chain Reaction (PCR) kit (Shanghai Kehua Bio-engineering Co., Ltd., China) at Beijing YouAn Hospital, Capital Medical University (the detection of HBV DNA ranged 500–10^8^ copies/ml).

### Outcome Assessment

Infants positive for HBsAg were considered immunoprophylaxis failure and HBV infection. The non-responder, low-responder, medium-responder and high-responder were defined as the infant negative for HBsAg or anti-HBs<10 IU/L, anti-HBs 10–100 IU/L, anti-HBs 100–1,000 IU/L, and anti-HBs≥1 000 IU/L, respectively. The factors for immunoprophylaxis failure, the association between non- or low-response to the HB vaccination were also analyzed. Adverse events (AEs) or severe AEs were recorded.

### Statistical analysis

The database was established with EpiData 3.02. An independent double input method was used to ensure the quality of data. Characteristics of the infants in immunoprophylaxis failure group and non-failure group, infants with non-responder and responder were compared using Student's test for continuous variables and Chi-square or Fisher's exact tests for categorical variables. The one-way ANOVA test or the chi-square for R×C tables test was used for more than two groups, Mann-Whitney U test was used for the frequency distribution of different responders between different groups. The data were analyzed by using Statistical Package for Social Science (SPSS) for windows, Version 13.0 (SPSS Inc., Chicago, IL, USA). All tests were two-tailed with the risk set at 5%, and statistically significance was defined as P<0.05.

## Results

### Characteristics of Infants

A total of 621 infants were included in this study. Among them, 347 (55.9%) were born to mothers positive for both HBsAg and HBeAg, 409 (65.9%) were born to mothers with detectable HBV DNA. The demographics and baseline characteristics of the infants are shown in Table 1.

**Table 1:** Baseline characteristics of the infants.

Characteristics	No. (%) of infants (Total infants 621)
*Sex*	
Female	289 (46.6)
Male	332 (53.4)
*Gestation weeks*	
37–38	33 (5.3)
38–39	108 (17.4)
39–40	215 (34.6)
40–41	186 (30.0)
41–42	74 (11.9)
≥42	5 (0.8)
*Birth weight (g)*	
2500–3000	89 (14.3)
3000–3500	286 (46.1)
3500–4000	197 (31.7)
≥4000	49 (7.9)
*Singleton*	
Yes	615 (99.0)
No	6 (1.0)

Abbreviations: HBV, hepatitis B virus.

### The Immunization Outcome in the Infants

Of the 621 infants, 18 were HBsAg-positive and anti-HBs negative (immunoprophylaxis failure) at 7 months. The rate of HBV infection was 2.9% (18/621), all the immunoprophylaxis failure infants were born to mothers positive for both HBsAg and HBeAg. Nine infants (1.4%, 9/621) did not achieve a protective level (≤10 IU/L) of anti-HBs despite the use of three doses of HB vaccine and became non-responders. A total of 594 (95.7%) infants achieved anti-HBs≥10 IU/L and became responders. Of these 594 responders, 22 were low responders (anti-HBs 10 to 99 IU/L), 191 were medium responders (anti-HBs 100 to 999 IU/L) and 381 were high responders (anti-HBs≥1000 IU/L).

### Factors Associated with Immunoprophylaxis Failure

HBV infection by mother-to-child transmission (HBV immunoprophylaxis failure) occurred in 18 of the total 621 infants (2.9%), the other 603 infants were non-failure infants. In Table 2, possible factors related to immunoprophylaxis failure were analyzed. The results showed that maternal HBeAg-positive and mother with detectable HBV DNA were strongly associated with infant's immunoprophylaxis failure (P<0.001 and P = 0.002, respectively). Other factors such as ALT level before delivery, birth weight and gestation weeks were not significantly related to the failure of immunoprophylaxis.

**Table 2:** Factors correlated with immunoprophylaxis failure.

Variables	Failure (n = 18)	Non-failure (n = 603)	*P* value
*Demographic data*			
Sex (F∶M)	5∶13	284∶319	0.105
Han nationality	17 (94.4)	569 (94.4)	1.000
*Maternal Data*			
Age at delivery (years, mean ± SD)	27.1±5.2	27.8±4.2	0.513
HBeAg positive	18 (100)	329 (54.6)	<0.001
HBV DNA detectable	18 (100)	391 (64.8)	0.002
ALT before delivery (IU/L, mean ± SD)	17.2±8.6	19.8±24.4	0.652
*Perinatal factors*			
Gestation weeks (w, mean ± STD)	39.5±1.3	39.3±1.1	0.383
Birth weight (g, mean ± STD)	3292±406	3394±422	0.306
1 minute APGAR score	9.3±1.1	9.4±0.7	0.451

Abbreviations: HBV, hepatitis B virus; ALT, alanine transaminase; HBeAg, hepatitis B e antigen ; HBsAg, hepatitis B surface antigen.

### Characteristics of non-Responders

Of the 603 non-immunoprophylaxis-failure infants, nine infants became non-responders after the combined HB vaccination and HBIG injection, while 594 infants achieved protective level (anti-HBs≥10 IU/L) designated as responders. The demographic data, maternal data and perinatal data between the non-responders and responders were compared. The results showed that the maternal ALT before delivery, gestation weeks, birth weight and other factors were not associated with non-response to HB vaccination (Table 3).

**Table 3:** Characteristics correlated with response and non-response n (%).

Variables	Non-responders (n = 9)	Responders (n = 594)	*P* value
*Demographic data*			
Sex (F∶M)	3∶6	281∶313	0.619
Han nationality	9 (100.0)	569 (95.8)	1.000
*Maternal Data*			
Age at delivery (years, mean ± SD)	28.9±2.5	27.8±4.2	0.418
HBeAg positive	5 (55.6)	324 (54.5)	1.000
HBV DNA detectable	6 (66.7)	385 (64.8)	1.000
ALT before delivery (IU/L, mean ± SD)	24.2±15.5	19.8±24.5	0.589
*Perinatal factors*			
Gestation weeks (w, mean ± STD)	38.9±1.5	39.3±1.1	0.275
Birth weight (g, mean ± STD)	3283±451	3396±421	0.306
1 minute APGAR score	9.6±0.5	9.5±0.7	0.428

Abbreviations: HBV, hepatitis B virus; ALT, alanine transaminase; HBeAg, hepatitis B e antigen; HBsAg, hepatitis B surface antigen.

### Vaccination Responders and Their Maternal HBV Infection Status

**Figure 2:**
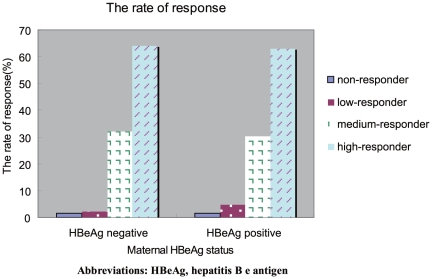
Distribution of different responders in mothers with different HBeAg status. Abbreviations: HBeAg, hepatitis B e antigen.

**Figure 3:**
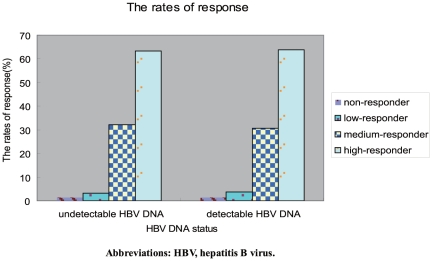
Frequency distribution of different responders in mother with different HBV DNA status. Abbreviations: HBV, hepatitis B virus.

We further divided the 603 infants who were non immunoprophylaxis failure into the following groups, 1) infants of HBeAg positive mothers, and 2) infants of HBeAg negative mothers; or 3) infants of detectable HBV DNA mothers, and 4) infants of undetectable HBV DNA mothers. Distributions of responders groups are shown in Figure 2 and Figure 3. Results indicated that the distribution of responders did not significantly differ between the maternal HBeAg positive group and maternal HBeAg negative group (P = 0.631), and between the mothers with detectable HBV DNA and without detectable HBV DNA (P = 0.912).

### Safety

Among the 621 infants, adverse events (AEs) were reported in five infants (0.8%). Two infants (0.3%) had injection side (right upper arm) reaction to the first dose vaccine, two infants (0.3%) developed fever, and one infant (0.2%) reported hives. There were no severe AEs attributable to vaccination.

## Discussion

To the best of our knowledge, this is the first report, showing a regimen of three doses of 10 µg HB recombinant vaccine plus two-dose of 200 IU HBIG, and with a 7-month follow-up for infants of HBsAg positive mothers. Our results showed most infants (95.7%) achieved protective level of antibodies (anti-HBs≥10 IU/L) at 7 months of age after receiving three doses HB vaccine and two-dose of HBIG.

In drastic contrast to placebo as control or non-intervention, administration of HBIG alone or vaccination alone significantly reduced hepatitis B occurrence [7]. In a report by Beasley et al., 172 infants of HBeAg positive HBsAg carrier mothers were given HBIG plus HBV vaccine and were followed for up to 2 years after birth. Overall, there was a significant difference in persistent HBsAg positivity in 6% (9/156) infants who were immunoprophylaxis failure, compared to 88% in historical controls [12]. Moreover, several previous studies showed the similar results: compared with sole HB vaccination or sole HBIG, combination of HB vaccine and HBIG significantly decreased the risk of perinatal transmission of HBV [7]. Therefore, the Centers for Disease Control and Prevention (CDC) in the US and the Advisory Committee on Immunization Practices (ACIP) both recommended administering immunoprophylaxis of HB vaccine and HBIg to those infants [13]. Similarly, the Chinese Society of Hepatology and Chinese Society of Infectious Diseases, and Chinese Medical Association also recommended the use of HB vaccine combined with HBIg for the infants of HBsAg positive mothers [14], [15].

Several previous studies had evaluated the immune outcomes of HB vaccine plus different doses of HBIG vaccination schedules in preventing perinatal transmission of HBV [11], [12]. In a double-blind randomized placebo-controlled study, Wong et al. compared four vaccination schedules in 140 infants by HBeAg-positive mothers, and found the persistence of HBsAg in infants was significantly lower in the three immunoprophylaxis groups (2.9% in receiving HB vaccine plus seven monthly HBIG injections group, 6.8% in vaccination plus one-dose HBIG injection group and 21.0% in HB vaccine alone group), compared with placebo control group (73.2%, P<0.0001). Moreover, the authors reported that vaccination alone was significantly less protective than vaccination plus multiple HBIg injections [11]. In a randomized double-blind, placebo-controlled efficacy trial of HBIG for prevention of the mother-to-infant transmitted HBsAg carrier state by Beasley et al. [16], HBIG was given immediately after birth to infants of e antigen positive HBsAg carrier mothers, and all infants were followed for at least 15 months. Among 61 placebo recipients, the carrier rate was 92%; compared with 26% among 57 infants who received 0.5 ml HBIG at birth, 3 months, and 6 months, and 54% among 67 infants who received a single 1.0 ml dose of HBIG at birth only. Efficacy was 71 and 42%, respectively, for the two treatment schedules. In a study by Grosheide et al. [17], for the infants born to HBsAg positive carrier mothers, an additional dose of 1 ml HBIG at 3 month of age did not increase the effect on prevention HBV transmission. However, transmission of HBV from one HBV infection mother to her infant is thought to occur generally the perinatal period, and the additional HBIG passive immunization should be given with one month after birth. Therefore, the multiple HBIG injections will not show significant additional benefit in preventing HBV perinatal transmission for the infants by mothers with HBsAg-positive when were given after one month of age.

Our results showed that immunoprophylaxis failure occurred in 2.9% (18/621) infants despite of receiving two-dose of HBIG combined with three doses HBV vaccination after birth. This immunoprophylaxis failure occurred more likely in infants of mothers positive for HBeAg and with detectable HBV DNA. These results are consistent with the previous reports [10], [18]–[21], indicating that the maternal HBV DNA and HBeAg positivity are the most important factors for the failure of immunoprophylaxis in infants of HBsAg seropositive mothers.

The rate of immunoprophylaxis failure (2.9%) in the current study is lower than that of previous reported, which were usually in the range of 5% to 15% [8]–[10], [19]. In a study in South Korea [10], 144 children who born to HBsAg positive mothers (81 mothers were HBeAg-seropositive and 64 mothers with detectable HBV DNA) received 100 IU HBIG and 10 mcg of plasma-derived HB vaccine or recombinant HB vaccine with 24 hours after birth followed by two further administrations of HB vaccine, and 17 (11.8%) children experienced immunoprophylaxis failure (HBV infection) after the completion of the HBV vaccination. The characteristics of the study pregnant women and the infants in this study were not different from the South Korea study, which concluded that maternal HBeAg and HBV DNA loadings are the risk factors for immunoprophylaxis failure.

In the majority of infants of HBsAg-positive mothers, immunization with vaccination can acquire protective levels of anti-HBs [7], [19]. However, there are still a few infants become non/low-responders. Previous studies showed the non/low-responders occurred in about 5–10% healthy individuals [22], [23]. In the current study, 1.4% (9/621) infants showed no response and 3.5% (22/621) infants were low-responders.

The mechanism of non-response to HBV immunization is unknown. The vaccine brand, age, male gender, obesity, and smoking in adult healthy people might be the associated factors [24], [25]. The study by Yen et al showed that male gender, age of older than 40 years and anti-HBc positive, were associated with non-response to HB vaccination [26]. Host genetic factors were also associated with non/low-response to HB immunization [27], [28]. In a study by Liu et al, non-response to HB vaccination in healthy individuals was associated with HLA B54 in Chinese [29]. Hsu et al reported the non/low-responses were associated with over-expression of HLA-DR14-DR52 in Taiwan [30]. In this study, all the participants were infants, and were given the same brand of HB vaccine, so the age, smoking and vaccine brands were not seemingly risk factors. Some other risk factors might be correlated with non-response to HBV immunization in infants, including delayed administration vaccination, preterm and low birth weight [19], [31]–[33]. In order to eliminate the influence of those factors, all infants included in this study had birth weight ≥2500 g, gestation ≥37 weeks, and were given the same HB vaccine within 6 hours of birth. The titers of anti-HBs will wane after vaccination during the follow-up [34], [35], so all the infants were tested HBV seromarkers at 7 months of age. Our results indicated the gender and birth weight were not correlated with immune response to vaccination.

There were a number of studies evaluating the immune outcomes of different vaccination schedules, different recombinant vaccines in infants of HBsAg positive mothers [7], [11], [19], [36], [37], but few studies evaluated whether maternal HBV infection status would influence the response to HB immunization in those infants. Our results showed that maternal HBeAg status and serum HBV DNA levels did not influence the response to HBV immunization in infants of HBsAg-positive mothers.

HB vaccination is well tolerated, and HB vaccination combined with HBIG injection is a well established safe regimen to treat mother to infant transmission. Most adverse events occurred were mild, while severe adverse events were rare [38], [39]. The adverse events in this study were similar to those of previous reports [38]–[41].

This study had a few limitations. First, the data were collected only from the infants who received the same immunoprophylaxis in one center and this may have selecting bias which could affect the quality of the study. Second, the HBV DNA levels in the infants at 7 months of age were not tested for identifying the HBV occult infection.

In summary, the present study showed that a vaccination by three doses HB vaccine plus two-dose of HBIG is effective way to prevent mother to infant transmission within the first seven months of birth. Maternal HBeAg positive and detectable HBV DNA status are associated with immunoprophylaxis failure, but not associated with non-/low-response to HB vaccination. Our results are preliminary, and the further study would focus on the follow-up of these infants until adolescence and evaluating their long-term immunological status.
